# Development and Validation of the Behavioral Tendencies Questionnaire

**DOI:** 10.1371/journal.pone.0140867

**Published:** 2015-11-04

**Authors:** Nicholas T. Van Dam, Anna Brown, Tom B. Mole, Jake H. Davis, Willoughby B. Britton, Judson A. Brewer

**Affiliations:** 1 Department of Psychiatry, Icahn School of Medicine at Mount Sinai, New York, New York, United States of America; 2 Nathan S. Kline Institute for Psychiatric Research, Orangeburg, New York, United States of America; 3 School of Psychology, University of Kent, Canterbury, United Kingdom; 4 Department of Psychiatry, University of Cambridge, Cambridge, United Kingdom; 5 Graduate Center, City University of New York, New York, New York, United States of America; 6 Department of Behavioral and Social Sciences, Brown University Medical School, Providence, Rhode Island, United States of America; 7 Departments of Medicine and Psychiatry, University of Massachusetts Medical School, Worcester, Massachusetts, United States of America; 8 Department of Psychiatry, Yale University School of Medicine, New Haven, Connecticut, United States of America; University of Vienna, AUSTRIA

## Abstract

At a fundamental level, taxonomy of behavior and behavioral tendencies can be described in terms of approach, avoid, or equivocate (i.e., neither approach nor avoid). While there are numerous theories of personality, temperament, and character, few seem to take advantage of parsimonious taxonomy. The present study sought to implement this taxonomy by creating a questionnaire based on a categorization of behavioral temperaments/tendencies first identified in Buddhist accounts over fifteen hundred years ago. Items were developed using historical and contemporary texts of the behavioral temperaments, described as “Greedy/Faithful”, “Aversive/Discerning”, and “Deluded/Speculative”. To both maintain this categorical typology and benefit from the advantageous properties of forced-choice response format (e.g., reduction of response biases), binary pairwise preferences for items were modeled using Latent Class Analysis (LCA). One sample (n_1_ = 394) was used to estimate the item parameters, and the second sample (n_2_ = 504) was used to classify the participants using the established parameters and cross-validate the classification against multiple other measures. The cross-validated measure exhibited good nomothetic span (construct-consistent relationships with related measures) that seemed to corroborate the ideas present in the original Buddhist source documents. The final 13-block questionnaire created from the best performing items (the Behavioral Tendencies Questionnaire or BTQ) is a psychometrically valid questionnaire that is historically consistent, based in behavioral tendencies, and promises practical and clinical utility particularly in settings that teach and study meditation practices such as Mindfulness Based Stress Reduction (MBSR).

## Introduction

Even at the level of a single-celled organism, behavior must necessarily fall into one of three possible categories: move towards (approach), move away (withdraw) or neither (no response). These three basic options cannot be reduced further and therefore would seem to represent the most parsimonious description of behavioral tendencies. Human beings share an evolutionary heritage, which has disposed us for certain common proclivities [1]. Variations on these general proclivities, along with differences in our adaptations to considerably more recent challenges (in evolutionary terms) have led to relatively stable (both across individuals and cultures), and largely behavioral, inter-individual differences, identified as dispositional traits [2]. While these tendencies may vary considerably across individuals and cultures and may comprise complicated constructs such as personality (e.g., [3]), temperament, (e.g., [4]), and character (e.g., [5]), at a basic level, these constructs would seem to build upon a tripartite behavioral categorization of approach, avoid, and neither approach nor avoid (i.e., inaction).

The focus on these basic behavioral tendencies is not meant to undermine the laudable efforts at characterizing human personality, as many theories have resulted in impressive results. For example, relatively recent work has shown that the Big 5 (Openness, Conscientiousness, Extraversion, Agreeableness, and Neuroticism), the most widely used categories of personality [3], show individual relationships to regional neuroanatomical volume [6] and intrinsic functional connectivity patterns of the brain [7]. However, the Big 5 may be characterized by two meta-traits, “Stability” and “Plasticity” [8], which may loosely relate to avoidance tendencies (stability—comprised of agreeableness, conscientiousness, and the inverse of neuroticism) and approach tendencies (plasticity—comprised of extraversion and openness).

One attempt to consider temperament/personality as tendencies for approach/avoidance was based in part on Gray’s neuropsychological model of anxiety [9]. Accordingly, Carver and White [10] developed a measure of the behavioral inhibition system (BIS) and the behavioral activation system (BAS), to which the fight-flight-freeze system (FFFS) has been appended in some conceptualizations [11]. While this early attempt to match personality to basic neurobehavioral tendencies is noteworthy, it is not without limitations. The original BIS/BAS [10] contained 13 items across 3 subscales related to appetitive motives (Drive, Fun Seeking, and Reward Responsiveness), but only 7 items on one subscale for avoidance. Further, the authors equated avoidance with behavioral inhibition (inaction) and fear, rather than the full range of behaviors commonly associated with the construct, including antipathy, repugnance, repulsion, dislike, hostility, anger, fear, and avoidance. Accordingly, the BIS items may reflect more fearful and neurotic tendencies than truly aversive ones, as reflected in strong correlations of the scale to negative affect, temperament, and harm avoidance [10]. Thus, while the BIS/BAS does reflect components of approach and avoidance, it may fail to capture essential pieces of avoidance. Further, it does not differentiate avoidance/aversion from the third fundamental option of inaction.

In addition to lacking basis in what could be considered the most parsimonious behavioral taxonomy (i.e., approach, avoidance, equivocation), most theories of temperament, personality, and character (e.g., [3–5, 12, 13]) are largely ‘Western’ in nature [3], potentially neglecting the cultural basis of meaning-making that forms the cornerstone of individual autobiographical representations [2]. Thus while existing models may meet aspects of criteria for one proposed evaluative system for testing personality theory (i.e., compatibility and predictive power); they may lack critical aspects of these criteria with regards to cross-cultural validity and parsimony [14, 15]. Further, because of their relatively recent conceptualization, current personality taxonomies have limited historical evidence to demonstrate their utility as a sustainably practical and cross-generational taxonomy tool.

### Buddhist Approach to Temperament

Unlike most contemporary theories of personality, which are predominantly Western, and less than a century old [16], a Buddhist theory of temperament [17], outlined in the *Visuddhimagga*, a 5^th^ century commentary on the early teachings of Theravada Buddhist doctrine, offers a non-western approach and one that has been in evidence for over fifteen hundred years (since at least the 5^th^ Century C.E.). Although age itself does not necessarily translate to better classification, the consistent use of such a system over time and cultures (see e.g., [17–19]) suggests the possibility of cross-generational validity. Further, the traditional text from which this typology is drawn characterizes the system not only as predictive of numerous aspects of behavior (private and public), but also, primarily, as a means to prescribe lifestyle advice. These lifestyles are described as maximally effective for encouraging the cultivation of skillful habits and offsetting unskillful habits [19, 20], a practice that continues among modern-day practitioners of various types of meditation practices [19], as well as yoga and alternative medical practices among others [18, 21]. This classification system is still in use and has been adapted by different cultures across different contexts. Specifically, the successful translation of mindfulness practices described in the Visuddhimagga (“The Path of Purification”) to modern evidence-based interventions suggests that other concepts from the same textual sources may also be amenable to empirical evaluation.

### The Buddhist Behavioral Temperaments

Buddhist theory posits that human beings react to pleasant experience by craving for its continuation, to unpleasant experience by craving for it to stop, and to neutral experience by ignoring it. Due to innate and learned dispositions, individuals are biased towards particular types of behavioral responses to stimuli. The Visuddhimagga refers to such temperaments using an Indic term, *cariya*, that translates literally as a ‘manner of going about’, or ‘manner of behavior’. This text presents its classification of behavioral types as a useful tool in tailoring interventions to individuals’ temperaments to achieve maximal benefit, specifying particular types of housing, clothing, and food, for instance, in addition to particular meditation techniques [17]. This may be one of the first descriptions of “personalized medicine”–matching an individual with a treatment based on the individual’s personal characteristics to improve outcomes.

The Buddhist classification of behavioral types considers numerous influences on how a person thinks, behaves, and feels, as well as basic organizational principles of the individual as an organism; the approach delineates these various influences in both external and internal factors. External signs include such factors as style of dress, postures and manner of walking, manner of eating, sleeping and waking, working style, and style of interacting with others. Internal signs mostly focus on the frequency of certain states of mind (e.g. anger, pride, etc.; more akin to many Western personality theories) [17, 19, 20].

The temperaments are characterized by three fundamental motivations (similar to those described above), greed (a pulling towards), aversion (a pushing away), and equivocation or confusion (characterized by neither pulling nor pushing but instead allowing the mind to wander either to disengage or escape from unsatisfactory conditions and/or to entertain tangential positive experiences related to satisfactory conditions). These three motivations correspond to three broad types of temperaments, which function as prototypes rather than exhaustive classifiers. In introducing the temperaments, the Visuddhimagga actually lists six types: greedy, faithful, aversive, discriminating, deluded, and speculative. However, the text suggests that the six types can be understood as forming three pairs of opposing properties corresponding to approach, avoid, and equivocate: 1) Greedy/Faithful type, the 2) Aversive/Discerning type, and the 3) Deluded/Speculative type. The two poles of each generally represent the skillful and unskillful aspects of each of these character types. For example, the greedy and faithful types, both manifest the same basic tendency, but the former in an unskillful way that leads to the perpetuation of and increase in suffering, whereas the latter manifests a skillful way that leads to the reduction of suffering ([17], p. 101–102). Each of these tendencies manifest in habitual ways, but importantly, also can be altered ([17], p. 102–3).

#### 1. Greedy/Faithful temperament (Approach Oriented)

A style of seeking pleasant, attractive, optimistic, beautiful, and enjoyable things and experiences characterizes the Greedy/Faithful temperament. In general, individuals of this type tend to be passionate, extroverted, spontaneous, and seek out novel opportunities and experiences. With an attentional bias towards the positive, these individuals tend to overlook faults or dangers. They are biased away from seeing their own faults, for instance, and thus may tend toward personal vanity. Equally, such positive biases can lead to a neglect of possible negative consequences (e.g. ‘selling’ an idea without responsibly mentioning possible downsides). On the other hand, this trait can manifest in positive ways, inclining individuals to be generous with their trust and with material gifts, and to take visionary leaps of faith necessary to move good works forward, saying ‘yes!’ when more detail-oriented individuals might consider all the ways in which things could go wrong.

Greed has also been characterized as unwholesome craving or desire. Predominantly greedy individuals could be characterized by always wanting more. “It desires fulfillment through pleasures, finding what it likes in the world of the senses. From liking, it can move quickly to craving, passion and sensuality” ([19], p. 137). If greed is a destructive and unskillful emotion motivating the pursuit of sensual objects, the corresponding quality of “faith”, here, is a motivation to pursue what is skillful and virtuous, “…greed does not give up what is harmful, while faith does not give up what is beneficial” ([17], p.102). As Gethin [22] puts it, “…Buddhist texts understand faith…not so much as a matter of intellectual assent to certain propositions about the world…[but]as a state of trust, confidence, affection, and devotion…” (p. 167). Thus while benevolence or generosity might be seen as the more precise opposite of greed, here benevolence is only one example of a virtuous quality that is motivated by faith. While affable, and often well liked, individuals of the greedy/faithful type often have difficulty with discipline, restraint, and commitment, and can become vain, self-centered, and unreliable while looking for the “best offer”. They are vulnerable to overindulging and addictions, sexual infidelity, and deception [17, 19, 20]. For additional discussion of the characteristics of the greedy/faithful type, especially behavioral descriptors, see Table 1.

**Table 1:** Features of three Buddhist temperaments described in the Visuddhimagga by different characteristics.

	TEMPERAMENT		
	*Greedy/Faithful*	*Aversive/Discerning*	*Deluded/Speculative*
**COGNITIVE FEATURES**			
**attentional bias**	positive; towards pleasant, overlooks faults/dangers	negative; towards unpleasant, overlooks pleasant/virtues	inattention or attentional bias away from present conditions towards distraction/mind-wandering
**skillful mind states**	trust; generosity; motivation to cultivate virtuous qualities	discernment; conscientiousness; prudence	equanimity; creativity
**unskillful mind states**	deceit, vanity, pride, self-centeredness, jealousy, avarice, and addiction	judgment, criticism, hostility, hatred, prejudice and ill-will, and aggression	negligence, confusion, doubt, paralyzing indecisiveness, speculation and inaction
**BEHAVIORAL FEATURES**			
**posture/movement**	graciously, with an elegant and springy step	stiffly and unevenly, with tension and tightness	hesitantly with a perplexed gait/ shuffle
**clothing style**	aesthetically arranged; neither too loose nor to tight	tightly	loosely; disheveled
**food preferences**	rich and sweet	sour and rough	no preference
**eating habit**	unhurriedly	hurriedly without savoring it	messily
**sleep habit**	comfortably	"with a scowl"	limbs sprawling or facedown
**morning awakening**	slowly	as if annoyed	with a "huh?"
**social interactions (meet new people)**	seize on trivial virtues and discount genuine faults; avoid conflict, even by dishonesty	come across as distant, tired, or bored; leave quickly as if anxious to go	copy what others are saying: confused, not knowing what to do or how to act
**response to novel environments**	notice whatever is pleasing, fixate upon it; leave pleasant circumstances slowly and with regret.	notice whatever is wrong; fixate on difficulties; seize upon any slightly unpleasant object; notice trivial faults and discount virtues	initially unaffected by pleasing or unpleasing features because unnoticed until others point out

In suggesting how to tailor a therapeutic intervention to persons of the greedy type, the Visuddhimagga advises providing conditions that counteract the tendency to grasp after sensual pleasures. Thus it is suggested that providing luxurious housing, clothing, and food is not beneficial for one who is predominantly greedy, and moreover that it is helpful to have personal interactions that discourage pride and vanity ([17], p. 107–8). For similar reasons, meditative contemplation of the body is especially suggested as a meditation object for those of a greedy type, including contemplation of bodily anatomy such as organs and fluids ([17], p. 113). This seems to be intended to counter attachment to the body, others’ as well as one’s own, and aimed ultimately at fostering a more balanced mental attitude.

#### 2. Aversive/Discerning temperament (Avoidance Oriented)

In contrast to the greedy type, the aversive type seeks to avoid or remove what is unpleasant, and therefore has an attentional bias towards faults, imperfections, burdens, and threats. Aversive types thrive in intellectual pursuits that require high levels of discernment, accuracy, and precision, and are often perceived as having high levels of competency or understanding. Where the greedy/faithful type is good at making the visionary leaps of faith necessary to move good works forward, aversive/discerning types excel at making detailed contingency plans to anticipate the many ways in which plans could go wrong. On a more negative note, however, these same qualities incline aversive types to judgment, criticism, hostility, hatred, prejudice, ill will, and aggression, all of which can lead to conflict and unpopularity. “The aversive temperament is constructed around judgment and rejection of experience…It is critical, quickly displeased, quarrelsome and disparaging of many things. Its quality of aversion can give rise to states of anger, vindictiveness, haughtiness, hatred, cruelty, aggression and struggle to control…” ([19], p. 174). Skillful and unskillful aspects are both recognized in the source text, which notes that whereas aversion is an unskillful and unwholesome motivation to find fault, discernment finds fault with those conditions that are not beneficial, in particular, unwholesome states of mind [17]. For additional discussion of the characteristics of the aversive/discerning type, especially behavioral descriptors, see Table 1.

In suggesting how to tailor a therapeutic intervention to persons of this type, the Visuddhimagga advises providing much more sensually pleasing conditions than those suggested for the greedy type. This text suggests aesthetically pleasing living quarters, with pleasing decorations, flowers, clothes, and perfumes, fine light clothing, as well as food that is inviting and “superior in every way” ([17], p. 108). The conditions suggested thus seem to be aimed at counteracting the tendency to aversion and of pushing away. Similarly, the development of loving kindness, compassion, sympathetic joy, and equanimity are especially recommended as meditation practices for one of an aversive temperament ([17], p. 114).

#### 3. Deluded/Speculative temperament (Equivocate)

While the greedy/faithful and aversive/discerning types are characterized by their strong motivational tendency of either grasping or pushing away, the deluded/speculative type is distinguished by the absence of a strong motivational tendency. It is characterized by a lack of awareness of present conditions, whether pleasant, unpleasant, or neutral. As a result, deluded/speculative types tend not to have strong immediate reactions and not to hold fixed opinions. On the positive side, this allows for creativity, being open to many possible options, and ‘thinking outside the box’. On the negative side, the deluded type can seem lost, constantly scattered, and prone to following the opinions of others because of uncertainty. Without an agenda, they can be laidback and easy-going, creative and “out of the box” pioneers. However, the lack of clarity, goals, or direction can lead to confusion, doubt, paralyzing indecisiveness, speculation, and inaction. “They seek to establish ease by ignoring what is happening or through dullness or inaction. The deluded temperament gives rise to perplexity and worry, doubt, negligence, scattered thoughts, anxiety and agitation.” ([19], p. 175). For additional discussion of the confused/speculative type, especially behavioral descriptors, see Table 1.

### Current Study

The current study aimed to develop a historically accurate and contemporarily applicable temperament questionnaire based on both traditional and contemporary texts, originating from the *Visuddhimagga* [17]. To counteract the effects of response styles and biases that can be particularly detrimental in cross-cultural research [23], we utilized a forced-choice response format, whereby items representing behaviors characteristic of the three types were compared. The psychometric properties of the questionnaire and specifically its ability to discriminate between the types were tested in two separate samples via Latent Class Analysis of choices made between pairs of items. Similar to Thurstonian Item Response Theory (IRT) modeling [24–27], this approach uses dummy-coded outcomes of rankings in each block of items for analysis. However, the measurement model assumes that categorical (latent classes) rather than continuous (latent traits) variables underlie the responses. To examine construct validity, we probed the relationship of the classes identified by this questionnaire to the predominant contemporary personality typology (i.e., the Big 5), as well as to approach/avoidance tendencies and behaviors, interpersonal tendencies, and decision-making styles.

## Materials and Methods

### Participants

A small sample of participants were recruited from the communities of Providence, RI and New Haven, CT (n = 36). All other participants (n = 859) were recruited through Amazon.com’s mechanical turk (MTURK) system. The MTURK works by creating a Human Intelligence Task (HIT), which are completed by an experimenter-selected number of potential participants from a pool of 500,000+ workers that use MTURK (see www.mturk.com). The experimenter creates an account to upload funds and create HITs. Reimbursement rate for a specified HIT is determined by the experimenter, often based on current norms among the MTURK for comparable amounts of time required to complete a HIT (see e.g., [28] for discussion of varying pay rates and its relatively small impact on the quality of data). Previous data analysis with MTURK has shown excellent internal consistency and good reliability, including high test-retest reliability [28]. Additionally, recent work has shown that data acquired via MTURK is not only equivalent to data collected in person, but also tends to provide more diverse demographics [29]. Data were collected in two waves. Data from the first wave were collected from a total of 394 individuals, 36 locally, and 354 from MTURK. Data from the second wave were collected from a total of 502 participants, all via MTURK. All procedures were determined to be exempt from institutional review board review due to the completely anonymous nature of the data collection.

### Measures

#### Behavioral Tendencies Questionnaire (BTQ)

An interdisciplinary team (comprising contemplative Buddhist scholars and/or practitioners, philosophers, clinical, social, personality, and other psychologists, as well as neuroscientists and psychiatrists) was formed to discuss questionnaire development. Appropriate source documents were first identified to develop a comprehensive representation of each of the different temperament types. After becoming familiar with the various descriptions of each of the temperament types, thematic analysis was performed and preliminary summaries of the temperaments were created using contemporary and classic texts (e.g., [17, 19]). Source documents consistently listed eight behavioral contexts (i.e. sleeping, waking, social interactions, etc.; see Table 1 for details). Individuals from the team drafted items for each behavioral category for each temperament type and consensus building was used to compile questions upon which the group agreed. The initial items were piloted on a small group for content consistency.

After the initial pilot, a set of 43 blocks of three items remained, each containing a stem phrase and three different possible responses, corresponding to each of the three temperament types. The response format for the BTQ was multidimensional forced choice, wherein participants were instructed to rank response options (i.e., 1^st^, 2^nd^, 3^rd^) based on how well each option characterized them for a given stem. Participants were instructed to rank the response option that was most like them as 1^st^ and the option that was least like them as 3^rd^ in each block.

#### Big Five Aspect Scales

The BFAS [12] is a 100-item measure of the ‘Big 5’ personality characteristics (openness, conscientiousness, extraversion, agreeableness, and neuroticism). Each ‘Big 5’ characteristic is comprised of two aspects: Openness = Intellect and Openness; Conscientiousness = Industriousness and Orderliness; Extraversion = Enthusiasm and Assertiveness; Agreeableness = Compassion and Politeness; Neuroticism = Withdrawal and Volatility. Each item is rated on a 1 (strongly disagree) to 5 (strongly agree) Likert-type scale. Past studies have revealed good psychometric properties and relationships to individual differences in brain structure [6, 12]. In the present sample, internal consistency for Big 5 (and domains) was as follows: Neuroticism, α = .906 (Withdrawal, α = .799; Volatility, α = .898); Agreeableness, α = .889 (Compassion, α = .897; Politeness, α = .776); Conscientiousness, α = .869 (Industriousness, α = .847; Orderliness, α = .811); Extraversion, α = .830 (Enthusiasm, α = .609; Assertiveness, α = .857); Openness, α = .869 (Openness, α = .839; Intellect, α = .814).

#### Behavioral Inhibition/Behavioral Activation Scales

The BIS/BAS [10] is a 20-item questionnaire designed to assess behavioral approach and avoidance tendencies. The behavioral approach system (BAS) is assessed across 13 items, while the behavioral inhibition system (BIS) is assessed across 7 items. Participants rate each item on a 4-point Likert-type response scale ranging from 1 (very true for me) to 4 (very false for me). In order to facilitate easy interpretation alongside other measures, we reverse-scored the scale such that higher mean scores indicate higher approach or inhibition. The BIS/BAS has shown generally good psychometric properties, with a 4-factor structure (BIS + 3 BAS subscales), and reliability/consistency values mostly > .7 in both nonclinical [10] and clinical samples [30]. In the present sample, internal consistency was as follows: BAS, α = .860 (Drive, α = .844; Fun Seeking, α = .776; Reward Responsiveness, α = .779); BIS, α = .854.

#### Conner’s Adult ADHD Rating Scales

The CAARS:S [31] is a 26-item, short-form assessment tool of Attention Deficit Hyperactivity Disorder symptoms. The items are rated on a 0 (not at all, never) to 3 (very much, very frequently), Likert-type response scale. The CAARS:S assesses four factor-derived subscales (Inattention and Memory Problems, Hyperactivity and Restlessness, Impulsivity and Emotional Lability, and Problems with Self-Concept), in addition to providing an inconsistency index. The inconsistency index consists of 16 items that are rated on a Likert-type scale ranging from 0 (not at all, never) to 3 (very much, frequently). The absolute difference for response pairs is summed and a cut-score is used. The index is an overall sum of absolute differences between pre-identified pairs of highly correlated items [31]. In the present sample, internal consistency was as follows: Inattention and Memory Problems, α = .840; Hyperactivity and Restlessness, α = .697; Impulsivity and Emotional Lability, α = .814; Problems with Self-Concept, α = .907.

#### Coping Responses Inventory

The CRI [32] is a 48-item questionnaire designed to assess approach and avoidance coping strategies in response to dealing with problems. The scale consists of 24 items that assess approach coping (subscales include logical analysis, positive reappraisal, seeking guidance and support, and problem solving) and 24 items that assess avoidance coping (subscales include cognitive avoidance, acceptance/resignation, seeking alternative rewards, and emotional discharge). Individual items are rated on a 1 (not at all) to 4 (fairly often) Likert-type response scale. The CRI has shown good predictive validity of psychological problems (esp. problem drinking) [33]. In the present sample, internal consistency was as follows: Approach coping, α = .890 (Logical analysis, α = .701; Positive reappraisal, α = .846; Seeking guidance and support, α = .637; Problem solving, α = .766); Avoidance coping, α = .850 (Cognitive avoidance, α = .890; Acceptance or resignation, α = .818; Seeking alternative rewards, α = .704; Emotional discharge, α = .514).

#### Life Orientation Test—Revised

The LOT-R [34] is a 6-item questionnaire (10 items if filler items are included) designed to measure optimism/pessimism. Three of the six items directly assess optimism and three directly assess pessimism. All items are scored so that higher scores indicate greater optimism. Individual items are rated on a 1 (I agree a lot) to 5 (I disagree a lot) Likert-type scale. In the present study, internal consistency was Cronbach’s α = .902.

#### Melbourne Decision Making Questionnaire

The MDMQ [35] is a 22-item questionnaire designed to assess coping patterns associated with the conflict theory of decision making [36]. Items are rated on a 0 (not true for me) to 2 (true for me), 3-point Likert-type response scale. Factor analysis supported a 4-factor structure comprised of the constructs of Buck-Passing (i.e., encouraging others to make difficult decisions), Hypervigilance (i.e., a hurried, anxious style of decision making), Procrastination (i.e., delaying/avoiding making decisions), and Vigilance (i.e., an exhaustive search style of decision making). The MDMQ has shown good psychometric properties across international samples [35]. In the present sample, internal consistency was as follows: Buck Passing, α = .891; Hypervigilance, α = .785; Procrastination α = .877; Vigilance α = .805.

#### Trust Inventory

The TI [37] is a 40-item measure of trust in others, generally (20 items) and romantic partners, specifically (20 items). Items are rated on a 1 (strongly disagree) to 4 (strongly agree), 4-point Likert-type rating scale. The scale has exhibited generally good psychometric properties [37]. Only the general trust items were administered in the present study. In the present study, internal consistency for the general subscale was, α = .708.

### Modeling Forced-Choice Data

A forced-choice response format has several benefits, including elimination of response biases acting uniformly across all items such as extreme responding and central tendency responding, and acquiescence [38], as well as minimization of exaggerated emotional coherence in assessments of different qualities (halo effect) [39]. Additionally, forced-choice formats eliminate the need for a rating scale, which assumes that all participants interpret that response option categories in the same way—often a problematic assumption (see e.g., [25]). However, forced-ranking items present unique challenges to data analysis. The primary problem is that if one considers means or sums of the rankings (e.g., 1, 2, 3), the values will be the same for each block (i.e., *M* = 2, *∑* = 6), regardless of the actual ranking. The resulting data has been called ordinal ipsative data (e.g., [40]) and requires complex analytic approaches (e.g., [41, 42]), with difficult to interpret results.

Fortunately, a solution to modeling multidimensional force-choice questionnaire data has recently been proposed [24–27]. This method, identified as Thurstonian Item Response Theory (IRT) allows for the recovery of latent trait structures underlying a response set [24–27]. Thus, for each ranking block of three items, all pairwise comparisons of items (i.e., A vs. B, A vs. C, B vs. C) are considered. Each pairwise comparison (say, A vs. B) is coded {A, B} = 1 if item A was preferred to B, and {A, B} = 0 otherwise. Hence, the three comparisons/contrasts (binary dummy variables) fully describe any given rank order of three items. For example, if a respondent assigns the rank orders A = 2, B = 1, and C = 3, the dummy coding will be {A, B} = 0, {A, C} = 1, {B, C} = 1. Because questionnaire items are usually designed to indicate some underlying trait, each pairwise comparison indicates two traits. Here we assume that items within a block indicate different traits. Thus, comparisons {A, B} and {A, C} provide information about the latent trait associated with A, comparisons {A, B} and {B, C} provide information about the latent trait associated with B, and comparisons {A, C} and {B, C} provide information about the latent trait associated with C. Detailed information about the development of these models and their implementation can be found in previous publications [24–27].

However, the questionnaire in the present study was designed not to indicate latent *traits* (continua along which participants may be placed), but latent *classes* (nominal types into which participants may be placed). These types (greedy, aversive and deluded) were hypothesized to underlie the observed preferences; hence, the measurement model suitable for the questionnaire is not an IRT model, but a Latent Class Analysis (LCA) model. This model is logically straightforward: each binary dummy variable resulting from pairwise comparison of two items is underlain by a categorical latent variable (latent class). Specifically, outcomes resulting from comparisons between Greedy and Aversive items (the first and the second items in each BTQ block, see S1 Appendix) are underlain by a latent categorical variable taking two possible values, or two classes—Greedy and Aversive. Those respondents who belong to the Greedy class, should tend to prefer Greedy items, hence their response to all item-pairs of this kind should be primarily {i_G_, i_A_} = 1. Conversely, respondents belonging to the Aversive class, should demonstrate primarily responses {i_G_, i_A_} = 0. The other two types of item comparisons are modeled in the same way. Thus, to explain the patterns of choices between items indicating Greedy and Deluded (first and third items in each block), a latent categorical variable with two levels is needed. Finally, choices between Aversive and Deluded items (second and third items in each block) are explained by a latent categorical variable with two levels.

In this study, we tested the three separate LCA models described above, which have an advantage of simplicity at the initial stages of developing and testing the questionnaire. Conditional on the latent classes, the pairwise responses within each model are independent; hence, all assumptions of a LCA measurement model are met to explore the optimal number of classes needed to describe the variability in responses, and assess the performance of individual items in measuring the intended classes. Due to local dependencies occurring between pairwise comparisons involving the same item (for example, {A, B} and {A, C}), and associated modeling challenges, we do not test all pairwise comparisons simultaneously in this study.

The LCA analyses were performed in MPLUS v7.2 [43], using the maximum likelihood estimator (robust). All other analyses were conducted in SPSS v20.

## Results

### Demographics and Data Screening

#### Demographics

Demographic details are provided in Table 2.

**Table 2:** Group Demographics.

	Sample 1 (n = 394^a^)	Sample 2 (n = 504)	
	M *(SD)*	M *(SD)*	*t*
**Age**	33.1 (12.9)	36.5 (12.6)	4.16***
	***% (n)***	***% (n)***	***χ*** ^***2***^
**Gender**			4.47*
% Female	63.7 (244)	58.2 (293)	
**Race/Ethnicity**			12.12*
% White	83.5 (320)	76.6 (386)	
% Asian	6.1 (23)	7.0 (35)	
% African American	3.3 (13)	8.3 (42)	
% Hispanic/Latino	2.5 (9)	3.9 (20)	
% Multi-racial	3.6 (14)	3.3 (17)	
% Other	1.0 (4)	0.9 (4)	
**Educational Attainment**			32.80***
% Graduate/Professional	14.7 (56)	11.1 (56)	
% College/University	43.9 (168)	41.1 (207)	
% Some College	38.3 (147)	33.5 (169)	
% High School	2.5 (10)	12.0 (65)	
% < 7 years	0.5 (2)	1.3 (7)	
**Work Status**			30.09***
% Full-time employed	39.1 (150)	49.0 (247)	
% Part-time employed	20.1 (77)	24.9 (126)	
% Unemployed > 1 month	15.5 (59)	13.1 (66)	
% Unemployed < 1 month	2.0 (8)	1.1 (6)	
Never employed	2.0 (8)	2.4 (12)	
Not in work force	21.3 (81)	9.4 (47)	

^a^ Data missing for n = 11.

Representative sample is n = 383.

***p < .001,

*p < .05.

#### Demographic comparisons

Within sample 1, there was no age difference between participants recruited locally and those recruited via Amazon.com’s Mechnical Turk, *t*(382) = 1.40, *p* = .16. Similarly, there was no difference in gender distribution, *χ* ^*2*^_(1)_ = 0.12, *p* > .5, racial/ethnic background, *χ* ^*2*^_(5)_ = 2.74, *p* > .5, marital status, *χ* ^*2*^_(5)_ = 0.80, *p* > .5, or household income, *χ* ^*2*^_(8)_ = 10.40, *p* = .24. There were, however, significant differences in educational level, *χ* ^*2*^_(4)_ = 21.89, *p* < .001, and occupational status, *χ* ^*2*^_(5)_ = 18.21, *p* < .01. The local sample had higher rates of completed graduate/professional training (36.1% vs. 12.6%), college/university completion (52.8% vs. 43.0%), but lower rates of partial college (11.1% vs. 41.1%). The local sample had substantially lower rates of unemployment (0.0% vs. 19.3%) and part-time employment (5.6% vs. 21.5%), but higher rates of full-time employment (63.9% vs. 36.6%) and not in labor force (27.8% vs. 20.7%). The rates of inconsistent responding, based on item pairs from the BFAS, were no different between groups, suggesting combination of groups would not be inappropriate.

#### Missing data

Among sample 1, there were 33 (8.6%) participants who completed less than 95% of all questionnaire items. These participants were excluded from further analyses. No participants from sample 2 had any missing data.

### Exploratory LCA Analyses in Sample 1 Data

The dummy coded binary contrasts in each model were explored fitting a sequence of LCA models with 1, 2, 3 etc. classes in sample 1, which contained responses to the full BTQ (43 blocks). Bayesian Information Criterion (BIC) was used to determine the best number of classes [44]. Exploring the between-class odds ratios of preferring the first option in each item-pair to the second option in calibration sample (sample 1) helped determine well performing items (items that discriminate well between the two classes) and flag poorly performing items. To this end, we used the recommended effect sizes for odds ratios (small = 1.5, medium = 3.5 and large = 9 [45]).

#### Greedy versus aversive

Dummy coded binary contrasts between the 1^st^ and the 2^nd^ item in each block were explored fitting a sequence of LCA models with 1, 2, 3 etc. classes. Table 3 reports the BIC and the entropy (index of classification quality) for each of the competing models. It can be seen that the 2-class solution was the best. The two classes were very clearly interpretable—the conditional probabilities of preferring the first item to the second in each pair were always higher in the first class. Hence, the first class represented the Greedy type (57% estimated prevalence), and the second represented Aversive (43% prevalence). Exploring the between-class odds ratios, two blocks showed large effect sizes, 12 blocks showed medium to large effect sizes, and 21 small to medium. Eight blocks discriminated poorly with effect sizes less than 1.5 (small).

**Table 3:** Goodness of fit of competing LCA models for Behavioral Tendencies Questionnaire (original 43-block version).

*Sample 1 (n = 394)*					
Model	# Classes	# parameters	Loglikelihood	BIC	Entropy
**G vs. A**	1	43	-11025.12	22307.21	
	**2**	**87**	**-10633.16**	**21786.27**	**.83**
	3	131	-10541.48	21865.86	.83
	4	175	-10454.94	21955.74	.82
**G vs. D**	1	43	-11047.06	22351.10	
	**2**	**87**	**-10746.22**	**22012.38**	**.75**
	3	131	-10638.07	22059.04	.82
	4	175	-10550.41	22146.69	.80
**A vs. D**	1	43	-10664.59	21586.17	
	**2**	**87**	**-10371.46**	**21262.87**	**.77**
	3	131	-10277.75	21338.41	.76
	4	175	-10188.06	21421.98	.81

*Note*: G = Greedy, A = Aversive, D = Deluded; BIC = Bayesian Information Criterion.

#### Greedy versus deluded

Dummy coded binary contrasts between the 1^st^ and 3^rd^ item in each block were explored fitting a sequence of LCA models. From Table 3 it can be seen that the 2-class solution was the best. The two classes were clearly interpretable as the Greedy type (58.2% estimated prevalence), and the Deluded type (41.8% prevalence). Exploring the between-class odds ratios, there were no large effects, 13 blocks showed medium to large effect sizes and 19 small to medium. Eleven blocks discriminated poorly with effect sizes less than 1.5 (small).

#### Aversive versus deluded

Dummy coded binary contrasts between the 2^nd^ and 3^rd^ item in each block were explored fitting a sequence of LCA models. From Table 3 it can be seen that the 2-class solution was again the best. The two classes were clearly interpretable as the Aversive (45.2% estimated prevalence) and Deluded types (54.8% prevalence). Exploring the between-class odds ratios, three blocks showed large effect sizes, 11 blocks showed medium to large effect sizes, and 14 small to medium. Fifteen blocks discriminated poorly with effect sizes less than 1.5 (small).

### Classification of Participants in Sample 2

After the item-pair parameters (i.e. thresholds) conditional on latent class membership have been established using sample 1, these parameters were used to calculate the conditional probabilities of belonging to latent classes for sample 2. Because sample 2 was administered a reduced version of the BTQ (with 29 blocks), only parameters for the available blocks were used. This calculation led to obtaining for each participant:

1. the probability of being Greedy from the model contrasting Greedy and Aversive, P_GA_(G), and the complementary probability of being Aversive P_GA_(A) = 1 –P_GA_(G);
2. the probability of being Greedy from the model contrasting Greedy and Deluded, P_GD_(G), and the complementary probability of being Deluded P_GD_(D) = 1 –P_GD_(G); and
3. the probability of being Aversive from the model contrasting Aversive and Deluded, P_AD_(A), and the complementary probability of being Deluded P_AD_(D) = 1 –P_AD_(A).

Thus, membership in each temperament class was based on the evidence from two separate models. Having these multiple evidences was beneficial since it allowed determining not only primary temperament/tendency type, but also secondary type. For instance, if an individual has the probabilities P_GA_(G)>P_GA_(A), P_GD_(G)>P_GD_(D) and P_AD_(A)>P_AD_(D), it must follow that his/her primary type is Greedy (since it was the most likely type compared to the alternatives), and his/her secondary type is Aversive. Given 3 classifications with 2 classes each, theoretically there should be 2^3^ = 8 perturbations of the probability inequalities. However, two perturbations out of eight are not admissible since they represent intransitive inequalities: (1) P_GA_(G)>P_GA_(A), P_GD_(G)<P_GD_(D) and P_AD_(A)>P_AD_(D) and (2) P_GA_(G)<P_GA_(A), P_GD_(G)>P_GD_(D) and P_AD_(A)<P_AD_(D). The remaining six inequalities are transitive and result in the following primary and secondary type combinations: GA, GD, AG, AD, DG, and DA. Considering only the primary type classification, out of all valid cases from sample 2, 42% were classified as the Greedy type, 32.6% as the Aversive type and 25.4% as the Deluded type. The primary type attributions were then used to validate the BTQ against other measures used in this study.

### Construct Validation with Sample 2

#### Sample 1: Relationships to Big Five

After classification of each participant into a primary temperament type based on the BTQ, the three groups were compared on scales and subscales of the BFAS. ANOVAs with Bonferroni corrected post-hoc tests were conducted on all 10 aspect scales and the Big Five scales. Group differences are reported in Table 4.

**Table 4:** Comparison of Groups in Sample 1 on Big Five Aspects and Traits.

		Greedy	Aversive	Deluded		**	*GvA*	*GvD*	*AvD*
		M *(SD)*	M *(SD)*	M *(SD)*	*p*	*Post-Hoc*	*Cohen’s d*		
***Big Five***	***Aspect***								
**Neuroticism**		2.69 (0.63)	2.81 (0.77)	2.99 (0.75)	.002	D>G	-0.17	-0.43	-0.24
	Withdrawal	2.75 (0.68)	2.86 (0.80)	3.09 (0.83)	.001	D>G	-0.15	-0.45	-0.28
	Volatility	2.62 (0.70)	2.76 (0.84)	2.89 (0.84)	.022	D>G	-0.18	-0.35	-0.15
**Agreeableness**		3.85 (0.61)	3.77 (0.52)	3.74 (0.56)	.215	-	0.14	0.19	0.06
	Compassion	3.97 (0.65)	3.73 (0.68)	3.77 (0.72)	.007	G>A,D	0.36	0.29	-0.06
	Politeness	3.73 (0.64)	3.80 (0.54)	3.71 (0.60)	.435	-	-0.12	0.03	0.16
**Conscientiousness**		3.47 (0.51)	3.68 (0.45)	2.92 (0.47)	< .001	A>G>D	-0.44	1.12	1.65
	Industriousness	3.51 (0.63)	3.54 (0.54)	2.94 (0.66)	< .001	G,A>D	-0.05	0.88	1.00
	Orderliness	3.42 (0.54)	3.83 (0.58)	2.91 (0.57)	< .001	A>G>D	-0.73	0.92	1.60
**Extraversion**		3.48 (0.44)	3.21 (0.48)	3.11 (0.56)	< .001	G>A,D	0.59	0.73	0.19
	Enthusiasm	3.54 (0.43)	3.13 (0.44)	3.11 (0.53)	< .001	G>A,D	0.94	0.89	0.04
	Assertiveness	3.42 (0.62)	3.28 (0.67)	3.10 (0.76)	.001	G>D	0.22	0.46	0.25
**Openness**		3.79 (0.59)	3.73 (0.49)	3.79 (0.58)	.660	-	0.11	0.00	-0.11
	Openness	3.78 (0.67)	3.52 (0.63)	3.80 (0.64)	.001	-	0.40	-0.03	-0.44
	Intellect	3.81 (0.61)	3.95 (0.63)	3.77 (0.72)	.102	A>G,D	-0.23	0.06	0.27

N.B. *p* values are from *F*-tests comparing all 3 groups.

Post-Hoc tests are Bonferroni corrected comparisons, where ‘>‘ indicates *p* < .05 and ‘,’ indicates *p* ≥ .05.

The greedy/faithful types were (relatively) highest on compassion (aspect scale of agreeableness) and extraversion (enthusiasm, and assertiveness, relative to the deluded/speculative type). The aversive/discerning types were highest on conscientiousness (overall, and orderliness aspect) and lowest on openness (aspect of Openness trait). Finally, the deluded/speculative types were higher than the greedy/faithful types (but not aversive/discerning types) on neuroticism (withdrawal and volatility aspects, as well as neuroticism trait) and lowest on conscientiousness (industriousness and orderliness aspects, as well as conscientiousness trait).

#### Sample 2: Relationships to Approach/Avoidance, Attentional Functioning, Interpersonal functioning, and decision making

Group differences were largely consistent with hypotheses (see Table 5). The greedy/faithful types were highest on overall approach orientation (BAS), and the drive and reward-responsiveness subscales of the BAS, the overall approach scale of the CRI, and positive reappraisal, and seeking guidance subscales of the CRI, the LOT-R (optimism), and the Trust Inventory. The aversive/discerning types were lowest on overall approach orientation (BAS) and the fun-seeking subscale of the BAS, lowest on the overall avoidance scale of the CRI, and significantly higher than the deluded/speculative types (but not the greedy/faithful types) on the vigilance subscale of the MDMQ. The deluded/speculative types were highest on all aspects of the CAARS (inattention and memory problems, hyperactivity and restlessness, impulsivity and emotional lability, and self-concept problems) as well as on the inconsistency index. They were also higher on cognitive avoidance, acceptance and resignation, and the emotional discharge subscales of the CRI, along with the overall avoidance scale of the CRI, as well as lowest on the overall approach scale of the CRI, and the logical analysis and problem-solving subscales of the CRI. The deluded/speculative types were additionally lowest on the LOT-R and highest on the Buck-Passing, Hypervigilance, and Procrastination subscales of the MDMQ.

**Table 5:** Comparison of Groups in Sample 2 on Behavioral Characteristics.

		Greedy	Aversive	Deluded		GvA	GvD	AvD
Scale	*Subscale*	M (*SD)*	M (*SD)*	M (*SD)*	*Post-Hoc*	*Cohen’s d*		
BAS		40.29 (5.80)	35.78 (6.06)	37.13 (5.67)	G>D>A	0.76	0.55	-0.23
	*Drive*	11.69 (2.78)	10.35 (2.63)	10.02 (2.38)	G>A,D	0.50	0.65	0.13
	*Reward Resp*.	17.27 (2.30)	16.08 (2.41)	16.22 (2.56)	G>A,D	0.51	0.43	-0.06
	*Fun-Seeking*	11.33 (2.37)	9.35 (2.60)	10.89 (2.43)	G,D>A	0.80	0.18	-0.61
BIS		19.23 (4.22)	20.06 (4.61)	21.70 (4.87)	D>G,A	-0.19	-0.54	-0.35
CAARS	*Inconsistency*	3.97 (2.52)	4.06 (2.18)	5.04 (2.31)	D>G,A	-0.04	-0.44	-0.44
	*Inattn/Memory*	8.23 (3.08)	8.31 (2.64)	12.29 (3.29)	D>G,A	-0.03	-1.27	-1.33
	*Hyper Restless*	9.87 (3.09)	9.48 (2.76)	11.46 (3.05)	D>G,A	0.13	-0.52	-0.68
	*Impulsive/Labile*	7.91 (2.59)	7.77 (2.38)	10.12 (3.49)	D>G,A	0.06	-0.72	-0.79
	*Self-Concept*	9.40 (3.76)	10.68 (4.11)	13.66 (4.32)	D>A>G	-0.32	-1.05	-0.71
CRI-Approach		74.20 (10.14)	71.52 (10.21)	66.66 (9.78)	G>A>D	0.26	0.76	0.49
	*Logical Analysis*	18.73 (2.89)	19.13 (2.65)	17.90 (2.93)	G,A>D	-0.14	0.29	0.44
	*Pos*. *Reappraisal*	18.95 (3.31)	17.34 (3.66)	16.36 (3.78)	G>A,D	0.46	0.73	0.26
	*Seek Guidance*	16.95 (3.15)	15.64 (3.58)	14.96 (3.09)	G>A,D	0.39	0.64	0.20
	*Prob*. *Solving*	19.57 (3.00)	19.41 (2.70)	18.95 (3.03)	G,A>D	0.06	0.21	0.16
CRI-Avoidance		57.44 (10.53)	54.40 (10.07)	61.33 (9.10)	D>G>A	0.30	-0.40	-0.72
	*Cog Avoidance*	14.23 (3.81)	13.82 (4.11)	16.60 (3.87)	D>G,A	0.10	-0.62	-0.70
	*Accept Resign*	13.86 (3.37)	13.55 (3.48)	15.54 (2.99)	D>G,A	0.09	-0.53	-0.61
	*Seek Alter Rew*.	16.17 (3.55)	14.40 (3.20)	14.86 (3.20)	G>A,D	0.52	0.39	-0.14
	*Emot*. *Discharge*	13.18 (3.22)	12.62 (2.82)	14.32 (2.72)	D>G,A	0.19	-0.38	-0.61
LOT-R		22.14 (5.40)	19.71 (6.11)	16.65 (6.06)	G>A>D	0.42	0.96	0.50
MDMQ	*Buck Pass*	9.66 (3.21)	9.94 (3.46)	12.00 (3.51)	D>G,A	-0.08	-0.70	-0.59
	*Hypervigilance*	8.72 (2.49)	8.55 (2.50)	10.31 (2.84)	D>G,A	0.07	-0.60	-0.66
	*Procrastination*	7.48 (2.48)	7.38 (2.50)	9.84 (3.00)	D>G,A	0.04	-0.86	-0.89
	*Vigilance*	15.13 (2.41)	15.71 (2.26)	14.54 (2.72)	A>D	-0.25	0.23	0.47
Trust Inventory		61.68 (10.41)	55.90 (11.34)	54.82 (10.28)	G>A,D	0.53	0.66	0.10

N.B. All scales have been scored such that higher scores indicate higher levels of the primary construct.

All *F*-tests were significant at *p <* .001, except LOT-R, where *p* = .001. Post-Hoc tests are Bonferroni corrected comparisons, where ‘>‘ indicates *p* < .05 and ‘,’ indicates *p* ≥ .05.

### Development of the BTQ short form

Based on the odds ratios computed for all pairs in sample 1 (odds of preferring the first item in the pair for members of class 1 divided by odds of preferring the first item in the pair for members of class 2), we selected the best discriminating blocks from the original 43 blocks (13 in total) to create a short version of the BTQ (BTQ-SF). The BTQ-SF is provided in S1 Appendix. Conditional probabilities of preferring the first item in each pair are given in Table 6. Using conditional probabilities, the individual probabilities of belonging to each class may be computed, and individuals classified in one of the three classes. We compared the estimated class membership based on the full BTQ (43 blocks) and the short version (13 blocks). The shortened version yielded very similar classification (see Table 7), with the coefficient of agreement between the two classifications *kappa* = .798.

**Table 6:** Conditional probabilities of preferring first item in a pair for the BTQ-SF.

block	pairs {G, A}		pairs {G, D}		pairs {A, D}	
	Greedy	Aversive	Greedy	Deluded	Aversive	Deluded
**1**	.47	.21	.62	.28	.77	.60
**2**	.60	.47	.72	.29	.76	.33
**3**	.57	.27	.77	.32	.89	.40
**4**	.52	.07	.58	.31	.79	.58
**5**	.55	.08	.55	.22	.87	.48
**6**	.37	.10	.49	.15	.78	.51
**7**	.52	.18	.79	.46	.92	.59
**8**	.47	.22	.59	.17	.75	.31
**9**	.54	.17	.57	.21	.75	.39
**10**	.39	.08	.51	.21	.80	.61
**11**	.55	.29	.58	.21	.59	.24
**12**	.42	.08	.81	.42	.96	.68
**13**	.61	.25	.79	.43	.87	.61

**Table 7:** Classification of participants from sample 1 based on the BTQ (43 blocks) and the BTQ-SF (13 blocks).

	*BTQ-SF (13 blocks)*			
*BTQ (43 blocks* *)*	Greedy	Aversive	Deluded	*Total*
**Greedy**	143	6	13	*162*
**Aversive**	9	89	8	*106*
**Deluded**	12	4	110	*126*
*Total*	*164*	*99*	*131*	*n = 394*
Overlap (%)	87.2	89.9	84.0	

## Discussion

The goals of the current project were to establish a parsimonious temperament scale based on historic observations and considerations of habitual behaviors [17] that had 1) good psychometric properties, and 2) good nomothetic span (appropriate relationships to other self-report measures).

We purposefully retained the original forced-choice format, ranking 3 response options with a single item stem, corresponding to each of the behavioral tendencies. Retaining this format was important, as the items were designed to represent categorical options broadly relating to descriptions of what individuals of different types would do in different contexts [17, 19]. Latent Class Analysis of pairwise comparisons between items indicating Greedy and Aversive types yielded two classes, which clearly corresponded to the hypothesized types. The same was true for the contrasts between Greedy and Deluded, and between Aversive and Deluded types. Individual items were assessed for their ability to discriminate between the types; many items had favorable properties, showing medium to large or large effect sizes. While we created an item pool reflecting the many characteristics of the traditional temperaments (see Table 1), not all item domains had acceptable psychometric properties. For example, items about food preferences had poor discriminative ability. Overall, however, the items of the final scale (BTQ-SF, see S1 Appendix) generally reflected the broad characteristics related to these historic temperament types. The class membership derived from the estimated conditional probabilities of responses resulted in clear classification with one predominant behavioral tendency type, as well as a secondary type.

When examining the classification groups of the first sample in relation to the Big Five Aspect Scales, the Greedy/Faithful types were compassionate and extraverted, the Aversive/Discerning types were conscientious and closed-minded (opposite of openness to experience), and the Deluded/Speculative were somewhat neurotic and careless (opposite of conscientious). The second sample completed measures of approach/avoidance (BIS/BAS), attentional problems (CAARS:S), coping styles (CRI), optimism, decision-making styles, and trust. As hypothesized, the Greedy/Faithful types were the most Approach-oriented, with significantly higher BAS scores than both other types on 2/3 approach-oriented subscales and the total BAS score. The Aversive/Discerning types had the lowest approach orientation overall (BAS), while the Deluded/Speculative types had the highest behavioral inhibition (BIS) mean score. Given that the BIS measures behavioral inhibition (more akin to inaction and/or fear) rather than avoidance [10], it is perhaps not surprising that the Deluded/Speculative types were highest on this measure. While the CAARS:S was designed to assess symptoms of ADHD, the nature of subscales (inattention and memory problems, hyperactivity and restlessness, impulsivity and emotional lability, and problems with self-concept) is consistent with the Deluded/Speculative type, who exhibited the highest scale means for all these subscales. Of additional interest, the Deluded/Speculative types exhibited the highest mean inconsistency scores, which provides support for this typology as a behavioral indicator of equivocation and/or difficulty committing to a given response (pattern).

In terms of coping strategies, the Greedy/Faithful types were largely approach-oriented in terms of problem-solving while the Deluded/Speculative types were largely avoidance-oriented. While we characterized the Aversive/Discerning types as generally avoidance-oriented, these individuals are also generally logical and conscientious (see Table 1), thus it is reasonable to conclude that they may not necessarily avoid problems while the indecisive nature of the Deluded/Speculative types may lead them to avoid problems in a number of ways.

In terms of optimism, the Greedy/Faithful types were highest and the Deluded/Speculative types lowest. While it is hypothesis-consistent that the Greedy/Faithful types were highest, one might have expected the Aversive/Discerning types to be lowest on optimism. However, one possibility is that the general uncertainty and ambiguity with which the Deluded/Speculative types view the world that leads them to be somewhat more pessimistic. Consistent with our hypotheses, the Deluded/Speculative type had the highest levels of maladaptive decision-making, exhibiting high levels of buck-passing, hypervigilance, and procrastination. Also consistent with our hypotheses, the Greedy/Faithful types had the highest mean scores on the Trust Inventory. Overall, it would seem that the types derived from the BTQ would seem to be largely consistent with those identified by the early source text [17].

### Limitations

It is worth noting that the temperaments, as originally described, rely heavily on observable aspects of behavior [17]. Historical and contemporary assignments of a particular temperament type were often done by experienced individuals who had detailed (longitudinal) knowledge of the individuals they were categorizing [20]. The present study relied solely on self-report of behavioral tendencies, which may pose unique problems for a typology historically characterized by observable behaviors. Items which held up to psychometric analysis in the present study may not represent aspects of the constructs that are better measured by observer report, actual behavioral measure, etc.

### Future Directions

#### Similarities to attachment types

The three insecure types of attachment [46] map relatively well with the behavioral temperaments. The anxious attachment style is one of clinging and neediness, as well as concern about involvement with others, similar to the Greedy temperament. The avoidant attachment style is one of pushing others away, often with increased internally and externally directed anger, and efforts to be self-sustaining, similar to the Aversive temperament. Finally, the disorganized attachment style is one of volatility and distortion, lacking a clear pattern or basis, similar to the Deluded temperament. Future studies might examine whether different attachment styles (e.g., [47]) map onto the behavioral temperaments. Relationships between these two constructs would further establish construct validity and compatibility and might permit important elaboration of how an individual’s attachment style might influence his/her behavioral predispositions.

#### Similarities to theoretical neurobehavioral systems

Another important future project may be to examine relationships between the behavioral temperaments and the approach/avoidance systems as measured experimentally (e.g., [48]). The greedy/faithful type might be described as very approach-oriented, while the aversive/discerning type might be described as very avoidance oriented. The deluded/speculative type might be considered neither approach nor avoidance oriented as this type is fundamentally confused about whether to pull towards or push away. Accordingly, there may be interesting relationships between the behavioral temperaments and related neurobehavioral functions of approach and avoidance systems [49].

#### Similarities to other measures of temperament

Other temperament scales have been developed with the aim of characterizing human experience and proclivities. For example, the Adult Temperament Questionnaire (ATQ) has shown similarities to the Big 5 in that it both had the emergence of a 5 factor model and subsequently demonstrated a higher-order 2 factor model [4, 50]. The Temperament and Character Inventory is another measure that has proposed relationships to basic neurochemistry [5]. Given that the behavioral temperament questionnaire has potentially important relationships to other measures of temperament (especially the ATQ; see e.g., [50]), future studies might examine these possible overlaps. Since the ATQ has direct relationships to various attentional and self-regulatory systems, these findings could potentially elucidate underlying neurocognitive components of the behavioral temperaments. Relationships of the BTQ to these scales might provide further insight into the construct validity of the BTQ, as well as helping to generate hypotheses about the neurophysiological basis of basic habitual behaviors.

#### Clinical and practical utility

Finally, measures like the Behavioral Tendency Questionnaire may provide a much-needed framework to tailor behavioral approaches to treatment. Indeed, the text from which we have drawn the description of these types presents the typology primarily as a means to provide prescriptive practices in relation to each of the different temperaments. This suggests that recent initiatives among medicine to individualize treatment (e.g., [51]) can also be utilized in meditation training as well as other behavioral therapies. The behavioral temperament scale presented here may be useful for indicating who might respond best to what type of meditation techniques [19], body-based practices and lifestyle changes [18, 21]. We have noted, for instance, how the Visuddhimagga suggests that individuals who manifest behavioral characteristic of the aversive type will do best meditating in visually pleasing, beautiful settings, and should be given meditation practices such as developing loving kindness. In contrast, it is suggested that greedy types will find austere and even unpleasant conditions more conducive to practice, presumably because these counteract the tendency to focus on pleasant stimuli. Similarly, because of its function to cut off mind-wandering, mindfulness of breathing is suggested as particularly suitable for those with deluded and speculative tendencies [17] (p. 114).

Notably, this characterization of temperament/personality is quite different from the most commonly used approach (i.e., the Big 5). A great deal of psychopathology is associated with Neuroticism. While perhaps unintended, the classification of one as predominantly neurotic does not really permit much room for positive interpretation. Further, the proposed notion that these personality traits are stable over time implies that the neurotic individual may always be that way. In stark contrast, the behavioral temperament system promotes a positive and negative aspect of each temperament that can be modified through training. The greedy individual is also characterized as faithful, the aversive as discerning, and the deluded as speculative. These reflect skillful and unskillful manifestations of the same types of behaviors. Thus an individual characterized as one type can actively work to direct behaviors in the more skillful direction. Further, the nature of these simple classifications may serve to enhance an individual’s awareness of his/her oft-overlooked behavioral tendencies and serve as a basis for discussion. As the typology is relatively simplistic, recalling that one is predominantly of a particular type could help one to place effort towards being more skillful, especially in the company of others who may have known similar or dissimilar temperaments.

These types of individualized approaches may be relatively easily adopted in modern medicine, given the brevity and ease of use of screening tools such as the BTQ. Indeed, although the Visuddhimagga’s discussion envisions a teacher having ample time to observe a student’s behavioral habits in daily life, s/he recommends questioning the student as a primary means of determining temperament [17] (p. 107). Moreover, given the origins of the scale, the BTQ may offer high cultural acceptability and relevance to the increasing population of Buddhists in Western societies. In the modern clinical context, individuals might fill out a screening BTQ form before beginning practice with a teacher or taking a mindfulness-based clinical intervention (e.g. Mindfulness-Based Stress Reduction; [52]), allowing the teacher to tailor instruction accordingly. Using this empirically derived approach to tailoring treatments or practices to individual temperaments may improve the efficacy of current treatments, though specific clinical trials are warranted to test such hypotheses.

## Conclusions

The present study aimed to develop and validate a behavioral tendencies questionnaire based on habitual behavior that was true to the traditional Buddhist texts in which mindfulness practice is described, has present day validity and applicability, and that seems to have practical utility in both scientific and clinical settings. The findings suggested good psychometric properties and construct consistent nomothetic span. Given the strong Buddhist influence on the BTQ, the measure would seem to have potential implications for individualizing meditation and mindfulness-based practices. Additional work is required to identify the extent to which the BTQ is a useful predictor of observable behavior, task performance, and neurobehavioral activity, but the present findings suggest that the scale has considerable potential.

## Supporting Information

S1 Appendix Behavioral Tendency Questionnaire. (DOC)
