# Supplementary material for: Development and Validation of the Behavioral Tendencies Questionnaire
Source: PLoS One. 2015 Nov 4;10(11):e0140867. doi: 10.1371/journal.pone.0140867 (PMC4633225; doi:10.1371/journal.pone.0140867)
Supplement: S1 Appendix — (DOC) [file pone.0140867.s001.doc]

| **INSTRUCTIONS:** This questionnaire contains a number of statements about tendencies that people have. For each item, select the 1 response option that is Most True for you, as well as the option 1 response option that is Least True response option that is for you. Note that you will only select 2 options for each item. |  | **Most True**  **for Me** | **Least True**  **for Me** |
| --- | --- | --- | --- |
| 1. If I were to plan a party, … | |  |  |
| …I would want it to be high energy, with lots of people. | | ☐ | ☐ |
| …I would only want certain people there. | | ☐ | ☐ |
| …it would be last minute and freeform. | | ☐ | ☐ |
| 2. When it comes to cleaning my room, I… | |  |  |
| …take pride in making things look great. | | ☐ | ☐ |
| …quickly notice problems, imperfections or untidiness. | | ☐ | ☐ |
| …don’t tend to notice or get bothered by clutter. | | ☐ | ☐ |
| 3. I prefer to make my living space … | |  |  |
| … organized. | | ☐ | ☐ |
| … creative chaos. | | ☐ | ☐ |
| … Beautiful. | | ☐ | ☐ |
| 4. When doing my job I like to … | |  |  |
| … consider future possibilities / wonder about the best way forward. | | ☐ | ☐ |
| … be passionate and energetic. | | ☐ | ☐ |
| … make sure everything is accurate. | | ☐ | ☐ |
| 5. When talking to other people, I might come across as … | |  |  |
| …affectionate. | | ☐ | ☐ |
| … philosophical. | | ☐ | ☐ |
| … realistic. | | ☐ | ☐ |
| 6. The disadvantage of my clothing style is that it may be… | |  |  |
| … unimaginative. | | ☐ | ☐ |
| … mismatched or uncoordinated. | | ☐ | ☐ |
| … decadent. | | ☐ | ☐ |
| 7. In general, I carry myself… | |  |  |
| … aimlessly. | | ☐ | ☐ |
| … briskly. | | ☐ | ☐ |
| … buoyantly. | | ☐ | ☐ |

Items are randomized and weighted. For scoring instructions and a scoring macro, please contact Dr. Brewer: judson.brewer@umassmed.edu

| **INSTRUCTIONS:** This questionnaire contains a number of statements about tendencies that people have. For each item, select the 1 response option that is Most True for you, as well as the option 1 response option that is Least True response option that is for you. Note that you will only select 2 options for each item. |  | **Most True**  **for Me** | **Least True**  **for Me** |
| --- | --- | --- | --- |
| 8. My room is … | |  |  |
| … neatly arranged. | | ☐ | ☐ |
| … messy. | | ☐ | ☐ |
| … richly decorated. | | ☐ | ☐ |
| 9. Generally, I tend to… | |  |  |
| … be in my own world. | | ☐ | ☐ |
| … have a strong desire for things. | | ☐ | ☐ |
| … be critical, but clear thinking. | | ☐ | ☐ |
| 10. At school, I might have been known for … | |  |  |
| …having lots of friends. | | ☐ | ☐ |
| …being intellectual. | | ☐ | ☐ |
| …day-dreaming. | | ☐ | ☐ |
| 11. I usually wear clothes in a way that is … | |  |  |
| … carefree. | | ☐ | ☐ |
| … fashionable and attractive. | | ☐ | ☐ |
| … neat and orderly. | | ☐ | ☐ |
| 12. I come across as … | |  |  |
| … thoughtful. | | ☐ | ☐ |
| … absent-minded. | | ☐ | ☐ |
| … affectionate. | | ☐ | ☐ |
| 13. When other people are enthusiastic about something, I… | |  |  |
| …jump on board and want to get involved. | | ☐ | ☐ |
| …might be skeptical of it. | | ☐ | ☐ |
| …go off on tangents. | | ☐ | ☐ |
